# Multi-omics: a bridge connecting genotype and phenotype for epilepsy?

**DOI:** 10.1186/s40364-025-00798-8

**Published:** 2025-06-18

**Authors:** Nan-Nan Wang, Fei Cao, Lin-Han Zhang, Yi-Fei Zheng, Da Xu

**Affiliations:** Department of Neurology, Union Hospital, Tongji Medical College, Huazhong University of Science and Technology, 1277 Jiefang Avenue, Wuhan, Hubei Province 430022 China

**Keywords:** Epilepsy, Multi-omics approaches, Single-cell omics, Spatial omics, Biomarkers, Drug target

## Abstract

Epilepsy is a collection of neurological disorders characterized by abnormal neuronal discharges, resulting in spontaneous and recurrent unprovoked seizures. Despite the use of over 20 anti-seizure drugs, conventional one-size-fits-all approaches are insufficient to meet the needs of all patients, and about 1/3 patients developed drug-resistant epilepsy. Recently, the establishment of precision medicine-based clinical management for epilepsy may bring new insights, especially omics-based approaches. Single omics approach is limited to addressing questions from a single molecular perspective. Whereas multi-omics approaches enable a comprehensive characterization of multiple molecules, revealing the complex molecular dysregulation networks underlying different epilepsy phenotypes. Furthermore, multi-omics methods have catalyzed a paradigm shift in scientific inquiry, transitioning from traditional hypothesis-driven types to data-driven research architectures. Despite the full potential of multi-omics research yet to be realized, its application in epilepsy holds great promise, from the discovery of epileptic biomarkers to personalized management. In this review, we performed a comprehensive overview of the omics technologies and multi-omics integration strategies, followed by an exploration of their role in enhancing the management of epilepsy treatment and care, hoping to provide new directions for future researches.

## Introduction

Epilepsy is a multi-factorial neurological disorder, characterized by heterogeneous etiologies including genetic, structural, immune, metabolic, infectious, and unknown factors [1]. Globally, more than 50 million patients are suffering from epilepsy, resulting in a huge burden to society and family [2]. Despite the availability of over 20 anti-seizure medications (ASMs), about 1/3 patients developed drug-resistant epilepsy [3]. Epilepsy surgery including surgical resection of epileptic foci and brain stimulation may be an alternation, but its high costs, precise localization of epileptogenic focus and surgical risks limited the widespread application. More importantly, the underlying mechanisms of the various types of epilepsy and seizures remain largely unknown, which are related to complex rearrangements at multiple levels, including gene, transcription, protein, and metabolism [4], highlighting the need for the identification of biomarkers to pinpoint high-risk patients for epilepsy development and relapse.

Omics techniques may be ideal tools for epilepsy research, which provide comprehensive assessments of different classes of biological molecules, such as DNA, RNA, and proteins in different species and individuals [5]. With the advent of high throughput sequencing and the decrease in sequencing cost, omics techniques have undergone a rapid expansion and is increasingly utilized in clinical settings. For example, the analysis of whole genomes elucidate previously unrecognized epilepsy-related genes and stratify patients with negative MRI results [6]. However, single omics approach fails to figure out the comprehensive complexity of the molecular regulatory networks involved in epileptogenesis. The integration of different omics technologies offer a comprehensive view of molecular complexity [7]. For instance, genomics identify candidate disease-causing genes for epilepsy, but it cannot quantify their expression levels [8]. The integration of transcriptomics can elucidate the spatiotemporal specificity of gene expression and its regulatory mechanisms. Novel multi-omics methods have shed light on the complex molecular dysregulation networks underlying specific epilepsy phenotypes [9]. For example, high-throughput multi-omics datasets accelerate the transition from hypothesis-driven to data-driven research approaches [10]. Besides, the emergence of single-cell omics provides cellular and molecular landscape rather than averaged data [11]. The inclusion of temporal and spatial factors enables the localization of the target site and elucidates the dynamic processes of epilepsy. In this review, we provide a comprehensive overview of emerging omics technologies in epilepsy, including their applications and limitations, as well as of recent advancements in multi-omics integration strategies, focusing on the potential of multi-omics technologies in precision health and clinical translation.

## Current state-of-the-art omics technologies

Omics technologies are defined as methods for probing and analyzing large amount of data to elucidate the structure and function at a particular level. Ever since the discovery of “Sanger sequencing” of DNA in 1977 [12], technologies for omics innovations have been developed by leaps and bounds. In recent years, genomics, transcriptomics, proteomics, and metabolomics have emerged as the cornerstone"four big omics"in medical research, as shown in Table 1. Additionally, the multifaceted nature of diseases necessities the development of supplementary omics technologies, including lipid-omics, immune-omics, radiomics, and ultras-omics. Previous omics studies primarily utilized chunky tissue samples, offering averaged measurements of multiple cells with limited resolution. Recent breakthroughs in cellular omics technologies have elucidated the interplay between intracellular and intercellular molecules with unprecedented resolution and scale. However, the utilization of omics technologies, including software for equipment, the establishment of databases, and the requirement for skilled technical expertise, elevates the temporal, human, material, and financial resource burdens, limiting their widely use.

**Table 1:** Comparisons of different omics technologies in epilepsy research

**Omics types**	**Research subject**	**Application**	**Advantages**	**Disadvantages**
**Single Omics**				
Genomics	DNA sequence, genetic and structural variation	Clarifying the genetic etiology of epilepsy	Comprehensive analysis of genetic information Technologically mature and cost-effective	Unable to directly reflecting gene expression or epigenetic regulation
Transcriptomics	mRNA, lncRNA, miRNA	Differential Gene Expression Analysis	Dynamically reflecting changes in gene expression and revealing regulatory networks	The abundance of RNA is not fully correlated with protein levels
Proteomics	Protein complexes and post-translational modification products	Discovery of epilepsy biomarkers and validation of drug targets	Directly linking functional mechanisms and revealing post-translational regulation	Limited technology and signal sensitivity
Metabolomics	Small molecule metabolites (< 1500 Da)	Diagnosis of epilepsy based on metabolic abnormalities in blood and cerebrospinal fluid	Real-time reflection of metabolic status Guiding dietary interventions and drug adjustments	Being susceptible to interference from diet and circadian rhythms Lacking standardized detection procedures
**Multi Omics**	DNA, RNA, Protein, Metabolites	The exploration of epileptogenic mechanisms, novel therapeutic targets, biomarkers, and development of personalized treatment regimens	Achieving the flow of data across different levels Avoiding the limitations of single-omics approaches and enabling the discovery of new biomarkers and mechanisms	Large volume of data, prohibitive costs, and data integration barriers

This table illustrates the scope of applications and the comparison of advantages and disadvantages of single omics and multi omics technologies

### Bulk omics technologies

Recently, the applications of spatial omics sparked heated discussions across various fields. Novel perspectives have been revealed to traditional bulk omics technologies, particularly in the field of transcriptomics, which have now been commercialized by 10 × Genomics as the Visium platform [13]. Therefore, the section on spatial omics will be introduced within the transcriptomics part.

#### Genomics

##### Basic characteristics

Genomics, including gene mapping, variant analysis, and comparative genomic analysis aimed to reveal the truth of genomes, expose DNA sequences and decipher the genetic information encoded in the genome [14], thus understanding the relationship between human diseases and genomic alterations. Currently, genomics is a mature field. It is surprising that the sequencing of the first human genome as a haploid reference took nearly 10 years, whereas the advent of next-generation sequencing has reduced this process into a five-day period [15]. Sequencing-based genetic testing approaches, such as whole-exome sequencing (WES) and whole-genome sequencing (WGS), are currently widely used in clinics. For example, the International League Against Epilepsy Consortium on Complex Epilepsies reported a genome-wide mega-analysis that identified 16 genome-wide significant loci, which encode ion-channel subunits, transcription factors, and a vitamin-B6 metabolism enzyme [16].

##### Optimized types

To meet different requirements, genomics has evolved into various classifications. For instance, GWAS is a pivotal tool in genomics to detect single-nucleotide polymorphisms (SNPs) correlated with epilepsy susceptibility, and to prioritize candidate genes, through comparing to the reference genomes [17]. However, most genomic studies are predominantly based on populations of European population [18]. To better capture the variation missed by using single reference genome, it is necessary to assemble a pan-genome, which contains all the DNA sequences information in a species. The pan-genome provides enhanced insights into presence/absence variation (PAV)-based genome-wide association studies (PAV-GWAS), which are vital in assessing population structure, analyzing diversity, and identifying important functional genes in humans [19]. The presentation of 3D and spatial genomics elucidates the organization of chromatin within the nucleus, detailing how the spatiotemporal structure of the genome orchestrates its unique features [20]; CRISPR functional genomics tools hold promise for elucidating gene function and regulation mechanisms, as well as for exploring how genes interact to influence complex disease traits [21]; Comparative genomics enable the comparison of genomes across different species, serving as a key to understanding evolutionary changes and adaptation among organisms [22]. Consider the case of COVID-19, comparative analysis of genomic data from various animal sources will be crucial in unraveling its origin, monitoring the emergence of new strains, and tracking pathogen transmission and evolution [23].

#### Metagenomics

Gut microbiota influences the brain's physiological, behavioral, and cognitive functions through alterations in gut microbial composition. Growing evidence has indicated that microbial communities were linked to neurological diseases, including epilepsy [24]. For example, researchers have indicated that individuals with epilepsy exhibit notable dysbiosis in their gut microbiota [25]. Probiotics could reduce nerve cell apoptosis and improve epilepsy symptoms by increasing the abundance of short-chain fatty acid (SCFA)-producing bacteriaRRG7 in the gut. Moreover, the gut microbiota can interact bidirectionally with orally administered ASM. On one hand, ASM and their metabolites can affect the gut microbiota microenvironment by modulating bacterial growth and composition. It has been reported that children with focal epilepsy were associated with the statistically differences in gut microbiota and carbohydrate metabolism, while these differences were reduced and the carbohydrate metabolism promoted after effective treatment [26]. On the other hand, ASMs can be metabolized by gut resident bacteria, altering their bioavailability, activity, or toxicity, which is a critical contributor to develop drug-resistant epilepsy [27].

Metagenomics research on gut microbiota is a valuable addition to enhance the research on epilepsy. For instance, metagenomic analysis in the feces collected 5 months after status epilepticus described a bacterial imprint associated with epilepsy, with the observed downregulation of lysine biosynthesis pathways, coupled with duodenal structural compromise [28]. This study presented new evidence of long-term alterations in the gut, as well as microbiota-related metabolic changes associated with epilepsy. In comparison to the collection of brain tissue, acquiring fecal samples is more convenient. Employing metagenomics to investigate the relationship between microbiota-gut-brain axis and the incidence of epilepsy presents a more viable approach. Furthermore, the integration of metagenomic techniques with other omics technologies can enhance the identification of microbial biomarkers linked to epilepsy for early diagnosis. However, the human genome is approximately one thousand times larger than the microbial genome, the presence of even a few human cells can entirely overwhelm the DNA components of microorganisms [29]. Enhancing sequencing depth and eliminating host DNA are potential strategies. Furthermore, like other omics technologies, metagenomics faces challenges in establishing causal relationships among alterations in fecal microbiota genes, which requires the development of new analysis and sequencing platforms.

#### Transcriptomics

##### Basic characteristics

RNA is a type of transient intermediary molecules, converting genetic information from DNA to proteins. Transcriptomics technologies enable comprehensive analysis of the entire set of RNA transcripts expressed in cells and tissues. The dynamic nature of transcriptomics reflects both the underlying genetic and epigenetic landscape, as well as environmental influences [30]. Although valuable, the mixed organization sacrifices crucial information by removing these tissues and cells from their native environments. Spatial information is frequently lost during tissue homogenization or dissociation. Immunohistochemistry (IHC) [31] and in situ hybridization (ISH) [32] represent the initial approaches for elucidating spatial information. However, these techniques only allow for the analysis of a limited number of genes and proteins at a time and are insufficient for omics-level measurements.

##### Basic introduction of spatial transcriptomics

Spatial omics enable the visualization and quantitative analysis of the full genome, transcriptome, and proteome with spatial distribution in tissue sections. Spatial transcriptomics has been the most extensively investigated technique within the omics field, recognized as the “Method of the Year” by Nature Methods in 2020 [33], and are increasingly being applied in clinical studies, as illustrated in Table 2.

**Table 2:** A summary of the applications of spatial transcriptomics approaches in clinical researches

**Methods**	**Disease**	**Sample**	**Discovery**
10 × Genomics Visium [34]	Epilepsy	Seizure mice models	Targeted CCL5 signaling therapy can attenuate neuroinflammation after seizure
GeoMx digital spatial profiling [35]	TNBC	Breast biopsies tissues	Eganelisib functions in tumor-associated macrophages reprogramming immunotherapy
10 × Genomics Visium [36]	HPV-negative OSCC	Tissues in tumor core and leading edge	malignant cells residing within the tumor core and leading edge possess unique transcriptomic profiles and ligand receptor interactions
ISC [37]	AD	Tissues around amyloid plaques	Demonstrating a gene co-expression network enriched for myelin and oligodendrocyte genes in the earlier phase of the disease
10 × Genomics Visium [38]	AIS	Brain tissues in ischemic hemisphere	Galectin-9 from microglia and macrophages to cell-surface glycoprotein CD44 is a critical signaling pathway after ischemic injury
NanoString GeoMx [39]	LS	Rectosigmoid mucosa	Observing a significant increase in the colonic mucosa levels of CD8 + T cells and natural killer
CosMx Stereo-seq [40]	AD	Amyloid plaque	The accumulation of microglia around plaques disrupts astrocytic signaling, destroying the balance in neuronal synaptic signaling

*TNBC* triple-negative breast cancer, *OSCC* oral squamous cell carcinoma, *ISC* in situ capture, *AD* Alzheimer’s disease, *AIS* acute ischemic stroke, *LS* lynch syndrome

Spatial transcriptomic methodologies can be primarily categorized as sequencing-based approaches and imaging-based approaches. Classical histological methodologies provide restricted spatial information, on which imaging-based spatial transcriptomics has progressively developed. Based on the methodology employed for RNA detection, imaging-based approaches can be classified into in situ sequencing (ISS), where transcripts are amplified and sequenced in the tissue directly with the aid of a probe ligation template [41], and in situ hybridization (ISH), where a target sequence is detected by the utilization of complementary fluorescently labelled probes [42]. Although offering high sensitivity and subcellular resolution, ISH-based and most ISS-based methods are targeted and exhibit limited sensitivity in detecting low-abundance unique genes, detecting a higher percentage of existing transcripts and requiring a priori knowledge of the genes of interest. Surprisingly, the advancement of technology optimized sequential fluorescence in situ hybridization (seqFISH +) can image mRNAs for 10,000 genes with high accuracy and sub-diffraction-limit resolution in the cortex [43]. Furthermore, scientists have proposed sequencing-based approaches to enhance gene throughput. Unlike imaging-based spatial transcriptomics, sequencing-based spatial transcriptomics enables the unbiased spatial barcoding of the entire transcriptome of tissue sections. The major innovation is the encoding of positional information onto transcripts before next-generation sequencing. Although useful, most sequencing-based approaches are unable to meet the requirements for sufficient resolution and sensitivity. The introduction of technologies such as DynaSpatia [44], Slide-seq V2 [45], and Stereo-seq [46] has led to a significant improvement in resolution. While the remarkable capabilities of spatial transcriptomics technologies are captivating, the pursuit of achieving the optimal balance between comprehensiveness and accuracy require further exploration.

#### Proteomics

##### Basic characteristics

The accumulations of mutations to a certain extent cause the anomalous manifestation of proteins, serving as candidates for biomarkers and key molecules. For example, the elevated level of AFP routinely indicates tumor diseases such as liver cancer and teratoma. Currently, proteomics has garnered significant acclaim for its applications in identifying and quantifying proteins present in a sample, as well as displaying the functional characteristics of proteins at various stages. Scientists can enhance other omics disciplines with essential information related to post-translational modifications, protein interactions, and protein localization [47]. For example, the prevalence of epilepsy is increased among Alzheimer’s Disease (AD) patients and patients with epilepsy are more likely to develop AD. It has been observed that 89% of proteins altered in the hippocampus of epileptics were also significantly altered in advanced AD, most of which are regulated by tau or interact with tau, highlighting the potential role of tau in regulating common pathways of both epilepsy and AD [48].

##### Commonly used techniques

The typical analytical procedures of proteomics are top-down and bottom-up approaches. The former directly analyzes and processes data from proteins, conversely, the latter works by enzymolysis of proteins to peptides [49]. In the past, many researchers focused on mass spectrometry (MS)-based proteomics, which functions through mass spectrometers to measure ion-mass-to-charge (m/z) values and signal intensities [49]. This method is limited in the number of proteins identified simultaneously. Following this, liquid chromatography tandem mass spectrometry (LC–MS/MS) technology emerged, combining the superior separation performance of liquid chromatography (LC) with the excellent sensitivity and selectivity of MS. Isotope-coded affinity tag (ICAT) labeling [50], isobaric tag for relative and absolute quantitation (iTRAQ) techniques [51], X-ray crystallography [52] and other approaches have recently developed for quantification and structural analysis. For example, proteomic analysis of the cortex and hippocampus in mouse models of post-traumatic epilepsy has revealed that post-traumatic epilepsy induces disruptions in pathways associated with mitochondrial function, post-translational modifications, and cellular transport [53].

#### Metabolomics

##### Basic characteristics

As the terminal downstream product of the genetic central dogma, almost all genotypic and protein alterations are presented as metabolites. Metabolomics, defined as a comprehensive and systematic identification and quantification of small molecule metabolites (< 1500 Da) through modern analytical platforms in biological samples at a particular time, provides a closer reflection of the phenotype variation than other omics. Although the origins of metabolomics can be traced back to ancient Greece, where urine colors, tastes, or smells, caused by metabolic alterations, were tested to diagnose diabetes [54], the advanced metabolomics methods were developed during the last two decades. Contrary to other omics, metabolomics is still not mature enough, and there are fewer metabolome databases publicly available.

##### Advanced approaches

Currently, major metabolomics approaches include targeted metabolomics, untargeted metabolomics, and widely targeted metabolomics. Targeted metabolomics attempts to identify and quantify a limited number of specific metabolites, which is an ideal method for biomarker detection, such as biochemical indicators commonly encountered in clinical testing. Untargeted metabolomics aims at acquiring data from all measurable analytes in a sample without bias. For widely targeted metabolomics, it combines the generality of untargeted methods with the accuracy of targeted methods. Advanced analytical instruments, such as LC/MS, gas chromatography-mass spectrometry (GC/MS) and non-destructive nuclear magnetic resonance (NMR) spectroscopy, facilitated metabolite profiling under complex bioinformatics conditions [55]. Despite still being in the initial stages, metabolomics has an influential role in exploring underlying mechanism, metabolite phenotyping and biomarkers identification. For instance, an untargeted metabolomics study demonstrated a glaring difference in the peripheral blood concentration of free fatty acids, glutamine, bilirubin, and iron metabolites between epilepsy patients and normal individuals [56], showing the potential to be serum biomarkers for patients with epilepsy.

#### Radiomics

Microscopic changes accumulating to a certain extent is likely to render structural and functional somatic changes, which can be detected by medical imaging analysis. Medical imaging enables the full-scale mapping within or around regions of interest via non-invasive approaches, bridging the gap between phenotypic and microscopic scale information. Radiomics accomplishes the translation of medical images, obtained from CT, PET, or MRI, into high-throughput mineable data and automatically extracts decisive features to supplement the estimation of clinical indices in various diseases [57]. In the past, the novel technology was more widely adopted in cancer diagnosis, clinical decision support, and prognosis judging. As radiomics approaches further developed, their application was expanded to other diseases, such as epilepsy. For example, juvenile myoclonic epilepsy (JME) and generalized tonic–clonic seizures alone (GTCA) present a common clinical profile, with a similar age of onset, the presence of generalized tonic–clonic seizures, and comparable electroencephalogram (EEG) background [58]. These overlapping characteristics can lead to misdiagnosis even among specialist neurologists. Therefore, scientists proposed MRI-based radiomics model and demonstrated its potential to diagnose as well as classify JME and GTCA through radiomics features from 1581 radiomics features [59]. This radiomics model had an AUC of 0.767 for the test set when differentiating between JME and GTCA.

Medical imaging remains challenging to accurately extract quantitative features from imaging results. Advancements in computational hardware and machine learning algorithms have made this accomplishment possible [60]. Additionally, radionics can make a still image of a moving data. A recent evolution in radiomics, known as delta analysis or delta texture radiomics, accounted for changes in structural features at different acquisition time points, typically before and after therapy [61]. The novel radiomics was booming in displaying the progress of diseases to determine whether the intervention was working and guiding adaptive treatment strategies based on its predictive capabilities and acquire.

#### Other advanced omics technologies

In addition to the commonly used technologies mentioned above, there have also been special types of omics proposed in recent years, including lipid-omics and immune-omics. Lipids are cellular metabolic products, in contrast to the majority of water-soluble cellular metabolites, the unique physical and chemical properties distinguish lipid-omics as a distinct discipline from general metabolomics. Glycosylation is a widespread form of molecular modification in the body, characterized by a highly diverse set of co- and posttranslational modifications of proteins and lipids. Approximately half of all proteins are glycosylated. High-throughput sequencing technology transformed our comprehension of the glycan chain diversity in biological organisms and their functions in life processes. This technology has also been extended to immunology and imaging, contributing significantly to these fields.

### Single-cell technologies

Traditional omics technologies may mask essential properties specific to diverse cellular subsets, and are insufficient for a comprehensive understanding of a specific disease. Single-cell technologies have revolutionized traditional bulk omics by providing the reference maps of the whole human body at unprecedented scale and resolution, especially in nervous system. For example, nearly one million glia spans among different neurological disorders, and different subtypes of cells can furnish potential markers for the diagnosis at the transcriptome level [62]. In the field of epilepsy, single-cell cellular indexing of transcriptomes studies have demonstrated the extensive activation of microglia and infiltration of pro-inflammatory immune cells in epileptic lesion tissues [63].

**Fig. 1:**
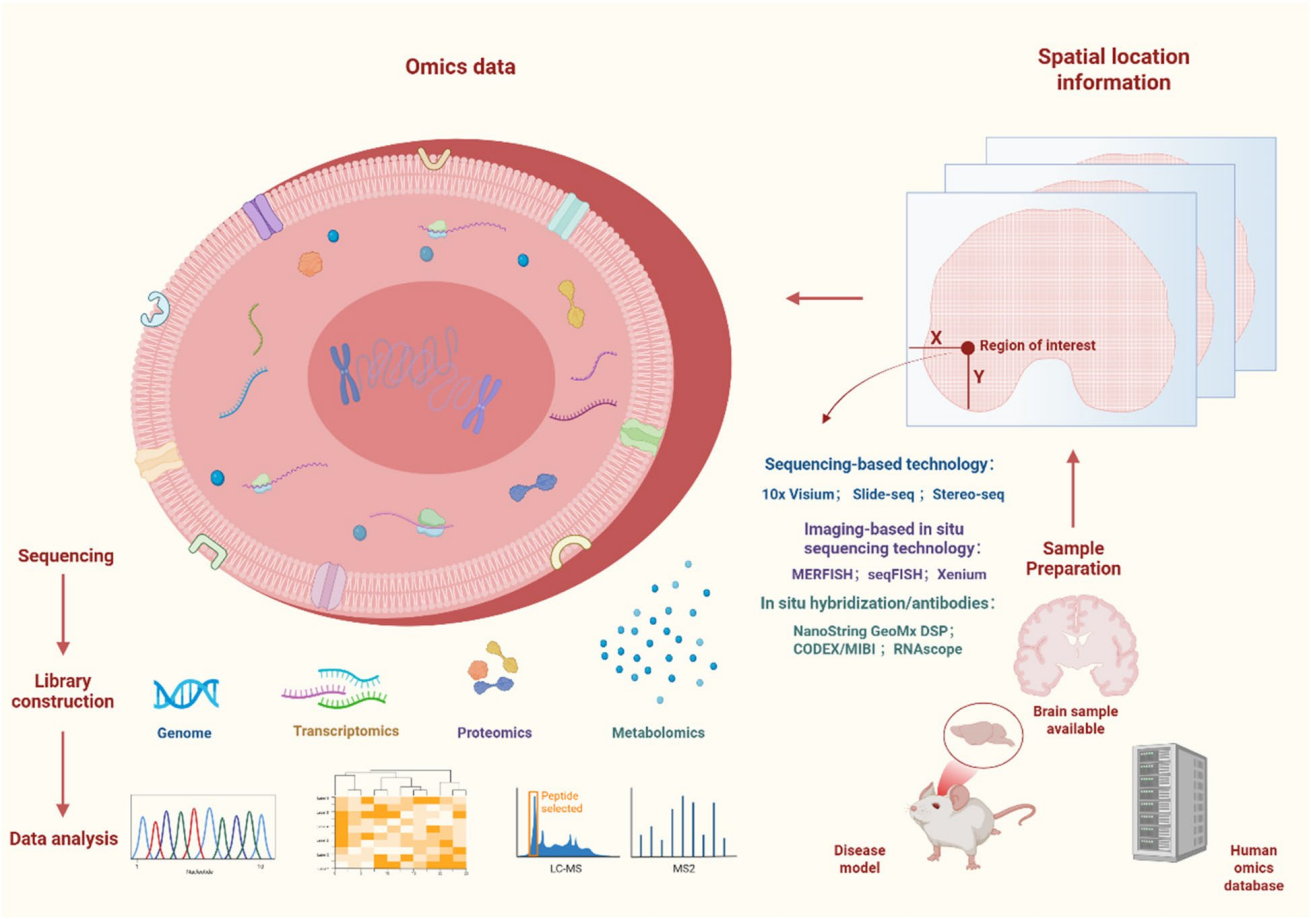
Brief workflow of spatial multi-Omics technology

Similar to spatial transcriptomics, the integration of single-cell technologies and spatial transcriptomics facilitated the identification of spatially restricted enrichments of different subtypes of cells in various regions (Fig. 1). Since the development of diseases is dynamical, the inclusion of a temporal variable is crucial for discovering the source and essence of diseases. Currently, multiple spatiotemporal analysis on diseases or organ development at single-cell resolution have emerged, as shown in Table 3. However, it is undeniable that this technology is not free from disadvantages. For instance, the human hippocampus, contains thousands of cells. Post-mortem neurotypical hippocampi from 32 subjects yielded profiles of 224,464 nuclei and 1,083 genes, with an average of 1,893 unique molecular identifiers (UMIs) per nucleus [64]. The volume of data is substantial, requiring professional bioinformatics analysis. Therefore, most studies only examined sample sizes ranging from a few to dozens, limiting the study's representativeness for the general population. Moreover, spatiotemporal transcriptomics represents the most demanding of all omics studies, its implementation is constrained by technical complexity, laborious experimental workflows, and high costs. With ongoing development, this technology will become widely utilized, much like genomics, which was once inaccessible, is now being progressively implemented in clinical settings.

**Table 3:** The application of advanced spatiotemporal transcriptomics in disease and human development

**Disease or research targets**	**Research contents**	**Outcomes**
The development progress of the human intestine [64]	Characterizing intestinal morphogenesis and cell types across time, location, and cellular compartments	(1) Identifying 101 cell states in intestine and their dynamic changes (2) Describing neural vascular, mesenchymal morphogenesis, and immune population of the developing gut (3) Pinpointing the origins of gut-associated lymphoid tissue and describing location-specific immune programs
The glia diversity in the human hippocampus [65]	Analyzing transcriptome atlas of the human hippocampus and spatiotemporal heterogeneity of glia	(1) Identifying subpopulations of glia and revealing their functions, association, and disease relevance Identifying dysregulated genes and pathological processes in specific glia subpopulations in AD
Human cerebellar development [66]	Depicting an integrative spatiotemporal landscape of human fetal cerebellar development systematically	(1) The combinations of transcription factors and cis-regulatory elements have a decisive role in in governing progenitor differentiation and cell fate determination (2) Granule cells located in different regions exhibit distinct molecular signatures
Pancreatic neuroendocrine tumor [67]	Exploring spatiotemporal heterogeneity of pancreatic neuroendocrine tumors, revealing the underlying mechanism for malignant progression	(1) Discovering the intra- and inter-heterogeneities of cell subpopulations in tumor tissues (2) Detecting several hallmarks of carcinogenesis activated at different stages of tumor progression (3) Developing a gene signature and defining the metastatic
The development of human heart [68]	Exploring the process of cardiac morphogenesis in human	(1) Obtaining spatiotemporal overview of gene expression dynamics collected at 4.5–5, 6.5, and 9 weeks (2) Mapping cell-type-specific gene expression to specific anatomical domains
Cervical carcinogenesis [69]	Deciphering the mysterious mechanism of persistent HPV infection induced during disease progression from normal to cervical cancer	(1) Identifying three HPV-related epithelial clusters (2) Founding that Treg/TH17 imbalance was associated with the change from persistent HPV infection to precancerous lesions (3) Verifying a homeostasis-balance-malignancy change during disease progression
Alzheimer’s disease [70]	Providing a comprehensive cellular map of disease progression	Demonstrating a core–shell structure where microglia closely contact amyloid-βplaques in the core, while astrocyte-like cells and oligodendrocyte precursor cells are enriched in the outer shells
Traumatic brain injury [71]	Profiling the tissue and cellular heterogeneity in hippocampus, frontal cortex, and blood leukocytes at different timescales	(1) Describing 24 distinct cell clusters representing observable gene expression differences (2) Verifying astrocytes as a key regulator of rehabilitating cell coordination, and mt-Rnr2 as a potential target for intervention (3) Demonstrating the application potential of humanin in reversing cognitive impairment

This table introduces several advanced spatiotemporal transcriptomics techniques, highlighting their exceptional ability to uncover key molecular mechanisms in various diseases and organ developmental processes, as well as their potential to develop new therapeutic technologies

## Multi-omics

Although single-omics technologies have attained considerable sophistication, molecular profiling restricted to a single biological layer does not effectively capture the dynamic and multifactorial nature of disease mechanisms. A comprehensive synthesis of data across molecular hierarchies is essential. Therefore, we will introduce the importance of multi-omics approaches in unraveling biological complexity in the following section.

### Background introduction

The physiological and pathological change represents an extremely complex condition, while single omics approaches are often inadequate. Multi-omics technologies represent a paradigm-shifting framework that synergizes heterogeneous molecular datasets, spanning genomic, transcriptomic, proteomic, and metabolomic dimensions, to decipher the intricate molecular interactomes and dysregulated signaling cascades of diseases [72]. This integrative strategy transcends the reductionist constraints of conventional single-omics studies, enabling causal reconstruction of genotype-to-phenotype trajectories across spatiotemporal scales.

Since the groundbreaking integration of yeast genomics and transcriptomics data by Gavin and colleagues [73], multi-omics studies have proliferated throughout biological research. For example, while the role of immune/inflammatory responses in epileptogenesis has garnered scientific consensus, traditional research has predominantly focused on isolated molecular targets (e.g., IL-1β or TNF-α). Multi-omics technologies now facilitate comprehensive profiling of 386 inflammation- and immune-related genes, enabling systematic investigations of their associations with epilepsy subtypes, therapeutic responses, and neuroinflammatory pathways [74].

### Multi-omics integration approaches

**Fig. 2:**
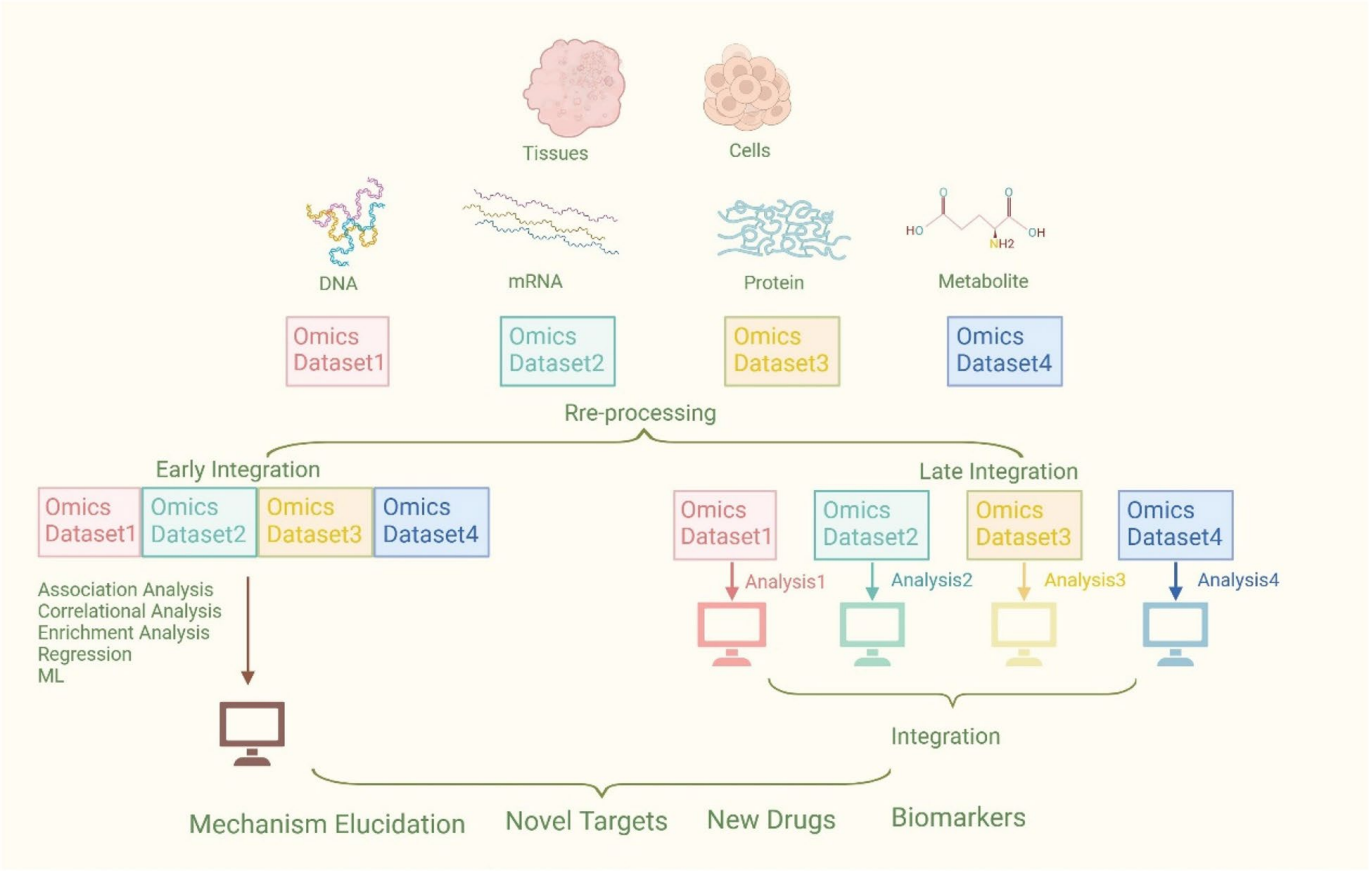
Main steps and potential applications of multi-omics technologies

Multi-omics technologies achieve the flow of information between biological layers (Fig. 2). However, without proper data processing, these data are enigmatic. Analyzing and integrating data, and extracting useful information generated from diverse platforms and large-scale heterogeneous samples, remains a significant challenge. In this section, we will delve into several advanced methodologies for integrating multi-omics data, and different types can be classified based on their emphasis.

#### Based on the relationship between different omics data

Based on the relationship between different omics data, integration methods can be divided into horizontal and vertical methods. Horizontal data integration refers to the integration with the same modality, obtained from different time points or different experiments [75]. This form of integration is mostly designed for a comparison between the two-class scenarios such as cases versus controls to identify concordant shared genes or other products from various modalities as anchors. Jian Zou et al. proposed a statistical framework, called Mutual Information Concordance Analysis (MICA) [76], to detect biomarkers across multiple omics studies. Nevertheless, the primary focus of this integration method remains on the same omics data, despite their origin from various experiments.

Instead of analyzing the same omics data from different samples, vertical data integration is dedicated to the exploration of different omics data types derived from the same samples [75]. This approach is advantageous for uncovering anchored modules that are shared across different molecular levels among the same disease or individual. Different sequencing platforms and molecular backgrounds makes it challenging but a major focus for current research, the specific methods will be detailed in the subsequent text.

As mentioned above, horizontal integration methods appeal anchored features from multiple samples, while vertical integration methods require anchored modules through feature conversion to align different modalities. However, different types of omics data typically do not share the same features, resulting in unpaired data. In this context, diagonal integration methods, which do not require modalities or anchored modules [77], offer a distinct advantage in integrating unpaired data and expanding the scope of possible data integration. For instance, scConfluence was a single-cell diagonal integration method that combined uncoupled autoencoders on the complete set of features with regularized Inverse Optimal Transport, possessing powerful ability to integrate unpaired data in various scRNA-surface protein and scRNA-scATAC integration problems [78].

Additionally, recently proposed mosaic integration methods can achieve any combination of horizontal, vertical, and diagonal integration. Imaging a grib consisting of m types of modalities and b batches, where each modality and batch contributes to a unique subset of information to the overall dataset. Mosaic integration methods aim to integrate any arbitrary subset data of the grib [77].

#### Based on the sequence of integration and analysis

Differentiated by the sequence of integration and analysis, the integration methods can be broadly classified as early integration, late, mixed, intermediate, and hierarchical [79]. Early integration concatenated all omics datasets into a single large matrix prior to analysis. For example, Anna Harutyunyan and colleagues collected proteomic and metabolomic data from genetic absence epilepsy rat models and non-epileptic controls into a large matrix, and employed a multi-omics, network-based approach to explore the underlying pathogenesis of absence epilepsy syndromes [80]. This approach was straightforward to implement and allowed for the combination of variables from each omics dataset, despite limitations in identifying the specific data distribution of each omics dataset. In contrast, late integration applied various analytical methods separately to each dataset, through combining their respective predictions [81]. For instance, Amanda M Canto et al. divided the ventral hippocampal dentate gyrus (DG) and Cornu Ammonis 3 (CA3) into dorsal and ventral areas and performed label-free proteomics and RNA-seq in mesial temporal lobe epilepsy rat models and controls separately [82]. This method does not suffer from the assembly of different types of data but cannot capture inter-omics interactions.

Mixed and intermediate methods were capable of achieving a balance between early and late integration. They operate by converting each omics dataset into a more simplified representation or by outputting new, constructed representations that not only elucidate the interrelationships between omics but also are lower-dimensional and less noisy [79].

#### Specific approaches

With available data becoming broader and larger, ensuant technical challenges are how to handle heterogeneous data and tease out meaningful signals from noise. The processing approaches vary significantly, and the working principle of each method is distinct.

##### Statistical-based approaches

The process of integrating multiple omics data sets fundamentally entails the analysis of a substantial database, frequently encountering the challenges of high dimensionality and limited sample sizes. Traditional statistical methods, including Principal Component Analysis (PCA), serve as foundational approaches for dimension reduction. PCA [83] could map a set of high-dimensional data into a low-dimensional space, by transforming multiple interrelated variables into a new set of variables through linear combinations. For example, Ming-Juan Wu et al. introduced an innovative model known as integrative hypergraph regularization principal component analysis (IHPCA) by incorporating hypergraph regularization constraints [84]. While achieving the unification of the data, it can also preserve the high-order manifold structure between the data, demonstrating stronger integration capability in sample clustering and common expression genes (co-expression genes) network analysis compared to other models. However, due to the mismatch in the data, PCA models may lose some detail information. Additionally, the constraints of PCA in achieving cross-omics consistency, causal interpretation, and dimensionality scalability, hinder its adoption for advanced systems biology research. Therefore, scientists have developed advanced computational strategies, including correlation analysis [85], multivariate analysis [86], and systems biology modeling [87], which better capture cross-omics interactions and improve biological interpretability in complex systems.

##### Artificial intelligence

In clinical applications, non-omics data such as unique clinical characteristics, provide a comprehensive understanding of diseases. The challenge lies in how to integrate omics data with non-omics data. In recent years, AI-driven integrative methods have immense potential to systematically uncover the dynamic interactions and hierarchical regulatory logic within biological systems, through automatically efficient data dimensionality reduction and cross-omics predictive association analysis. For example, integrated multi-omics analysis of gut microbiome-derived metabolomic profiles and serum inflammatory mediators, powered by AI-driven methods, identified a predictive biomarker signature: elevated gut-specific bifidobacterium abundance coupled with heightened serum TNF-α levels is strongly associated with improved therapeutic efficacy of ketogenic diet (KD) in pediatric patients with drug-resistant epilepsy [88].

As a vital branch of AI, machine learning (ML), and in particular deep learning, was the most extensively employed methods, which were capable of identifying non-linear and hierarchical features within the data and subsequently integrating heterogeneous data types seamlessly. For instance, Haris Hakeem and coworkers validate a deep learning model using readily available clinical information to predict treatment success with the first ASM for patients with epilepsy [89]. The trained model achieved a weighted balanced accuracy of 0.62 (95% CI, 0.60–0.64) on the test set. ML methods can be broadly classified into supervised and unsupervised learning. Supervised learning employs labeled data to train a model for predicting or estimating the output of unknown data [14]. Conversely, unsupervised learning focuses on learning features and patterns from unlabeled data to discover the inherent structure and relationships within the data. Although the principles are different, these approaches are all capable of automatically capturing intricate patterns and making intelligent decisions based on data. For example, there was a report on the integration of multiple high-throughput omics datasets from Alzheimer disease cohorts, with clinical and neuropathological data using a Bayesian integrative clustering method [90]. This method allowed the identification of four distinct multimodal molecular profiles, which were not only associated with poor cognitive function but also indicated a more rapid progression of disease.

However, the inherent complexity of multi-omics data imposes fundamental limitations on all AI-driven approaches including state-of-the-art deep learning models, in achieving robust and biologically interpretable integration. Critical limitations arise from three interconnected factors. Firstly, most omics studies are constrained by small sample sizes (< 100 cases), which starkly contrasts with the large-scale annotated datasets required to train robust AI models. This mismatch heightens overfitting risks [91]. Besides, although contemporary AI algorithms demonstrate impressive capabilities in identifying statistical correlations within complex datasets, they are fundamentally limited in elucidating causative mechanisms. For example, Researchers utilized machine learning and integrative clustering techniques to analyze multi-omics data in order to identify alterations in seizure-related pathways among patients with AD. Nevertheless, their analysis was confined to detecting dysregulation in just 15 epilepsy-associated genes, including sodium/potassium channel genes such as SCN1A and KCNA2, and failed to clarify directionality or causal relationships [92]. The critical shortcoming hampers their effectiveness in clinical translation. Lastly, the integration of diverse omics layers results in ultrahigh-dimensional feature spaces, increasing computational complexity, while current computational infrastructure and algorithms are insufficient for the efficient processing of these multidimensional datasets. Therefore, continuous optimization of algorithms has become an urgent imperative.

## The application of multi-omics in epilepsy

Multi-omics technology stands as a powerful and cutting-edge tool in epilepsy research, enabling the exploration of epileptogenic mechanisms, identification of novel therapeutic targets, discovery of biomarkers, and development of personalized treatment regimens. However, the in-depth exploration remains challenging due to technical complexities, prohibitive costs, and data integration barriers.

### Main application

The maintenance of health requires overcoming challenges posed by growth, environmental factors, and physical demands. The disruption of perfect coordination and counterbalance may lead to the onset of various diseases, including epilepsy. Consequently, understanding the complexities of epilepsy necessitates multi-level biological analyses. In the following part, we will introduce the powerful application of integrated multi-omics methods in epilepsy.

#### Exploring the underlying mechanism

Research into the mechanisms of epileptogenesis has spanned over half a century [93], generating numerous hypotheses, including neural network hyperexcitability, energy metabolism dysregulation, neuroimmune-inflammatory cascades, and maladaptive synaptic plasticity. Nevertheless, these hypotheses partially explain the complex process of epilepsy. For instance, the IL-1 receptor antagonist Anakinra, targeting neuroinflammatory responses, appears to demonstrate significant therapeutic efficacy predominantly in the treatment of Febrile Infection-Related Epilepsy Syndrome (FIRES) [94], but demonstrating limited effectiveness in other epilepsy subtypes. Consequently, underlying mechanisms is essential to effectively treat epilepsy.

In comparison to conventional approaches, multi-omics technologies provide distinct advantages in revealing cross-hierarchical molecular interactions and mapping the spatiotemporal dynamics of epileptogenesis. Imagine a scenario where we generate multi-omics profiles and decipher their temporal transitions and spatial distributions during the disease progression from normal tissues to undiscovered precursor lesions to epileptic foci, through which we can elucidate the key genes and metabolic molecules. This has been achieved in a study proposed by Chong Liu and other researchers [95]. They performed transcriptomic and proteomic analysis of the hippocampus in epileptic mice and obtained 19 differentially expressed molecules at protein level and gene level after combined analysis. Eventually, they demonstrated that SerpinA3N in astrocytes promoted aggravated seizures by activating the NF-κB signaling pathway. Besides, Ye Peng and colleagues employed shotgun metagenomic sequencing in conjunction with untargeted metabolomic analysis to compare the gut microbiome and metabolome of 8 children with non-epileptic cerebral palsy (NECP) against those of 13 children with cerebral palsy with epilepsy (CPE) [96]. This study elucidated the potential roles of Bacteroides fragilis and Dialister invisus in neuroprotection. We are confident that the application of multi-omics will ultimately decipher the molecular mechanisms driving epilepsy development, paving the way for mechanism-targeted therapeutic innovations.

#### Searching for novel drug targets

**Fig. 3:**
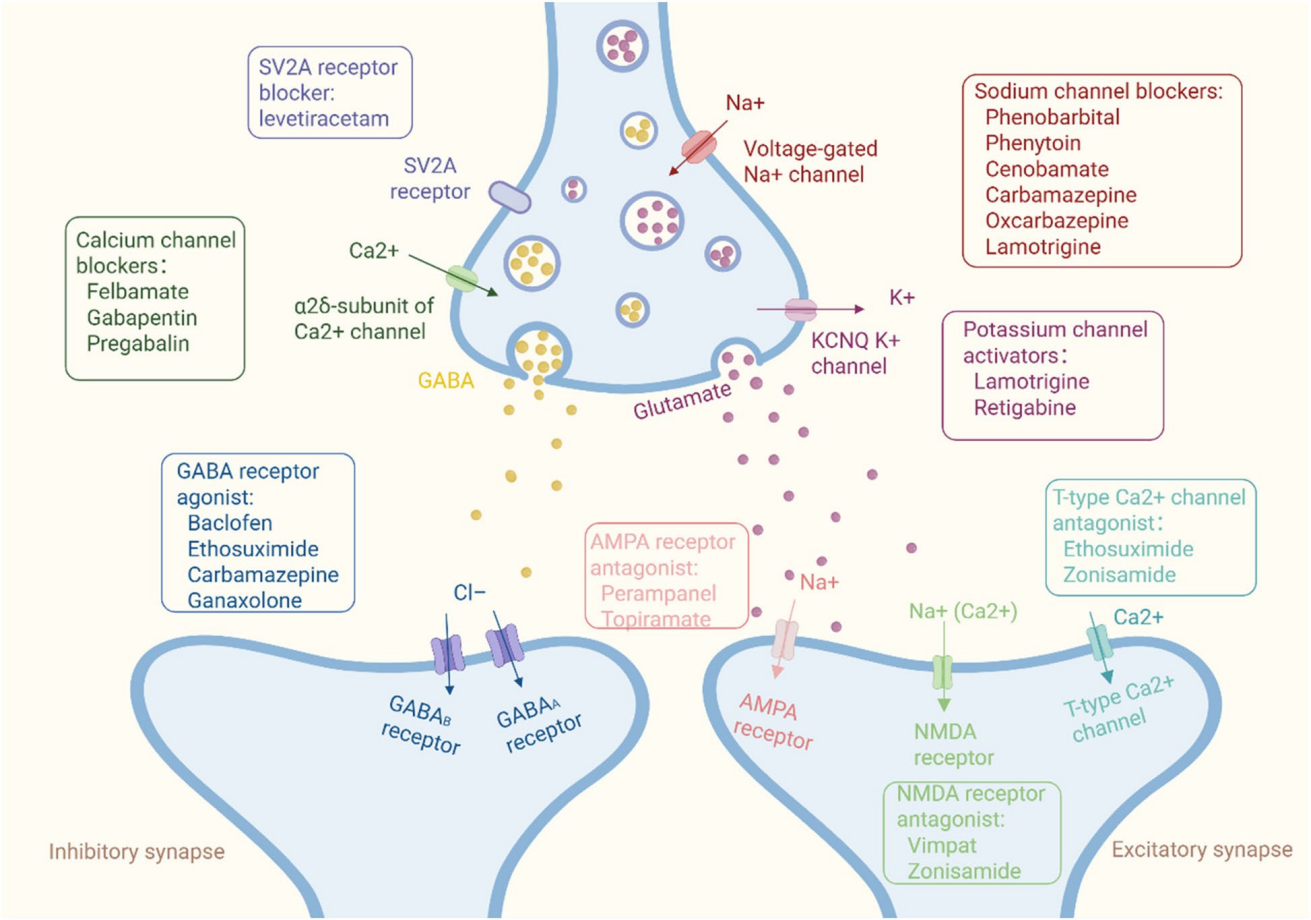
Common antiepileptic drugs and main target sites

ASMs constitute the cornerstone of treatment for epilepsy. Regrettably, most ASMs primarily target membrane ion channels and neurotransmission [97] (Fig. 3), only suppress seizure activity rather than addressing the underlying causes of the condition, acting as antiseizure drugs rather than antiepileptic drugs. Further complicating the situation, a substantial proportion of patients with epilepsy experience ongoing drug-resistance, with one-third of individuals still have uncontrollable epilepsy and develop resistance. The development of new drugs is urgent.

Multi-omics integration-based approaches could explore differential genes, specific molecules, and key molecular modules behind epilepsy, represents a promising approach for identifying novel drug targets and addressing drug resistance. However, the field still suffers from a paucity of validated success cases in epilepsy. Most researches were dedicated to using multi-omics methods to validate the therapeutic efficacy of novel drugs or to identify potential pathways associated with disease outcomes after intervention. For example, Pablo M. Casillas-Espinosa and colleagues conducted targeted and untargeted proteomic and metabolomic analyses to validate the efficacy of sodium selenate treatment in rats with chronic epilepsy [98]. Hongyuan Lu and colleagues employed transcriptomic and metabolomic analyses to assess the efficacy of cannabidiol (CBD) in the treatment of epilepsy [99]. This form of discovering new drugs is still"passive,"and the process of developing new drugs must transition from being"passive"to being"active,"which involves actively seeking out critical target molecules by delving into the underlying mechanisms. Despite the challenges, it is essential.

#### Discovering biomarkers for early identification

The success of a prevention program is largely influenced by the identification of individuals at high risk, which requires reliable biomarkers. Biomarkers are defined as objectively measurable variables of a biologic process, providing reliable information of individual in a specific moment. Seizure is characterized by abnormal conduction of neural electrical signals. Electroencephalography (EEG) is a valuable tool for identifying the state of the epileptic brain, which can not only confirm the diagnosis, but also clarify the type of epilepsy. Among all electrical signals, high-frequency oscillations (HFOs) are a potentially useful biomarker for identifying the seizure localization and comprehending the ictogenesis-related mechanisms in epilepsy [100]. However, HFOs are challenging to detect due to relative low signal-to-noise ratio, and cannot guarantee the accuracy and timeliness of the information provided. ECoG and intracranial electrodes can effectively reduce signal-to-noise ratios, but invasive electrodes are typically constructed from rigid metals and silicon, exhibiting a significant mismatch in mechanical properties with brain tissue and may result in severe inflammatory response and irreversible brain damages [101]. Furthermore, seizures are unpredictable, the ethical considerations of monitoring patients for EEG recordings without prior warning are controversial. Long-term video-EEG monitoring appears to be an effective solution, yet prolonged electrode wear for several months, may result in skin irritation or damage, causing discomfort, and is associated with a gradual degradation of the quality of the acquired electrical signals over time. The exploration of easily detectable and non-harmful biomarkers for epilepsy is vital.

Biomarkers derived from cerebrospinal fluid or brain tissue are considered the most reliable, however, their extraction is highly invasive and challenging to perform. Previous studies have explored the use of patient plasma(e.g., purine concentration) [102], feces (e.g., Firmicutes, Bifidobacterium, Streptococcus) [103], and other easily obtainable human tissues for biomarker detection, yet these approaches have not been widely implemented. The primary reason is insufficient specificity and lack of standardized detection methods. The application of multi-omics analysis to these samples can transcend the limitations of single approaches by identifying critical biomarkers within the context of molecular regulatory networks. For instance, Franz Huschner and colleagues employed whole genome sequencing, proteomics, and metabolomics [104], revealing that multiple molecules in blood including serum deoxycytidine monophosphate (dCMP) exhibited significant differences between the Tuberous Sclerosis Complex (TSC) group and the control group, potentially serving as biomarkers. Youssef Khalil and colleagues assessed the reliability of 6-oxopiperidinic acid as a biomarker for ALDH7A1 deficiency-related epileptic encephalopathy using urinary multi-omics analysis [105]. Nevertheless, the results obtained have not yet undergone comprehensive clinical validation, and findings from various laboratories remain inconsistent. The development of universally effective biomarkers necessitates ongoing exploration.

#### Personalized management

Epilepsy is a multifaceted condition with various risk factors. There are subtle variations in the genetic and phenotypic characteristics of epilepsy across different regions, among different populations, and even between individual subjects. Traditional one-size-fits-all treatment have not considered these variations. As the field progresses into the era of disease-modifying therapies, the individualized management of epilepsy is receiving more attention. However, these approaches require strict and meticulous technical methods to discover the uniqueness of the individual's disease presentation.

Multi-omics methods serve as the most comprehensive tools for uncovering individual differences. From this, doctors are capable of developing personalized treatment plans. However, this concept remains largely theoretical and has yet to be implemented. Considering the current deficiencies, these techniques are more frequently applied in researches of epilepsy families and genetic epilepsy. Moreover, current studies are insufficiently comprehensive, leaving new issues unresolved. For example, although Ainhoa Pascual-Alonso and her team discovered several genes that can act as biomarkers and therapeutic targets in MECP2 duplication syndrome-induced epilepsy through RNAseq and proteomics analysis [106], there is no further evidence to prove the effectiveness of these targets in the treatment of epilepsy. All biological and medical research is ultimately aimed at clinical application. Future research, while creating new omics technologies, should also prioritize the cross-disciplinary application of multi-omics technologies.

### Main application limitations and future research directions

Despite the formidable potential of multi-omics technologies, their application in epilepsy research and clinical translation remains limited.

#### In the field of basic research

The primary challenge lies in the complexity of data integration. Data from different omics are often not dimensionally compatible, complicating the alignment of data volumes. A meticulous standardization process is necessary to mitigate biases among various data types, but the unmet demands may cause inconsistencies in the outcomes, and may lead to false conclusions in some cases. For instance, Herbert Schulz and coworkers attempted to explore the pathogenesis of temporal lobe epilepsy [107]. They utilized multi-omics approaches to elucidate the cis-regulatory effects of single-nucleotide polymorphisms (SNPs) on DNA methylation (meQTL) and gene expression (eQTL) in the hippocampal tissues of 110 patients with temporal lobe epilepsy. Although they identified correlations between CpG methylation and RNA expression for 34 genes, the overlap between hippocampal eQTLs and meQTLs was associated with schizophrenia rather than with epilepsy. This scenario highlights the necessity for cross-omics standardization and probabilistic quantification, alongside the imperative for multidisciplinary collaboration to enhance computational algorithms.

Furthermore, the substantial amount of data imposes significant demands on both integration and analytical computation. While the present state of technological advancement is insufficient for conducting high-quality multi-omics research. For example, inadequate coverage of single-cell omics technologies may result in loss of important data and false-positive findings [108]. The application of artificial intelligence for data management is fundamentally characterized by a lack of transparency, often referred to as a"black box"approach. This obscurity complicates the extraction of reliable relationships between molecules [109]. These situations necessitate the ongoing optimization of sequencing modes and data processing procedures.

In terms of research design, most studies are retrospective, lacking prospective clinical validation, which may limit the interpretative power of their findings. However, the difficulty of clinical validation is substantial, and the outcomes of basic research, such as the discovery of biomarkers, often do not align with clinical outcomes, such as disease progression. During prospective clinical validation, it is crucial to establish a strong connection between test results and therapeutic interventions.

#### In the field of clinical translation

The potential for achieving clinical translation using multi-omics technologies is restricted. An important reason is that most samples in multi-omics studies are derived from epileptic animal models, while there is a substantial difference in the epileptogenic mechanism between humans and animals. Regarding studies that utilize human samples, most are constrained by small sample sizes and homogeneous study populations. The expression level of the same gene can vary significantly among individuals. Research that relies solely on the average results from a limited number of samples is insufficient for guiding personalized precision treatment. Establishing a global epilepsy omics database through multi-center collaboration is feasible.

The sequencing, mass spectrometry, and other technical platforms and analysis workflows employed by different laboratories are not consistent, resulting in difficulties in reproducing the results. Moreover, multi omics data often includes sensitive information of participants, such as genetic details and disease risks. Researches involving human may also adversely affect patients during prospective validation, requiring ethical considerations. Therefore, enhancing supervision throughout the entire research process is vital. On the one hand, implementing oversight in research process, establishing standardized guidelines, and requiring laboratories to establish rigorous quality control systems and global multi-center validation can effectively eliminate data deviations, promoting the industrialization and accessibility of the technology. On the other hand, regulatory authorities also play a crucial role in safeguarding patient safety and ensuring ethical compliance by conducting pre-approval safety assessments and ethical reviews.

Moreover, the clinical translation of multi-omics requires coordinated collaboration across multiple disciplines. However, differing perspectives among biologists, clinicians, and data scientists can create disconnects. For instance, basic researchers may prioritize technological advancements, whereas clinicians focus on the clinical applications, potentially leading to a misalignment between research objectives and clinical requirements. It is necessary to form a translational medicine team comprising clinicians, biologists, engineers, and data scientists [110].

Another non-negligible issue is that the cost of multi-omics monitoring technologies is prohibitively high. An excessive number of samples can impose a substantial financial burden, while an insufficient number of samples may compromise both the statistical power and the reliability of the conclusions. The complexity of the procedures and the challenges of training specialized personnel also impede the advancement of multi-omics research. Hence, the development of more cost-effective technologies is essential.

## Conclusions

Multi-omics approaches, renowned for their ability to uncover associations between various molecules, have been increasingly applied in epilepsy research. Compared to conventional detection technologies, multi-omics approaches provide higher resolution and more comprehensive detection capability, enabling the identification of key molecules that might have been previously overlooked. Moreover, they offer a more thorough understanding of the complex processes underlying the onset and development of epilepsy.

Although multi-omics approaches hold a bright prospect in the further in-depth researches and better management of epilepsy, they still face many challenges and uncertainties, making the clinical implementation of multi-omics difficulty. Firstly, multi omics generates a vast amount of data, the computation, analysis, and integration of which are extremely complex requiring dedicated equipment, trained technicians, and sufficient financial reserves, which are not available in all laboratories. Secondly, multi omics data are frequently heterogeneous, characterized by the presence of thousands of variables, which exacerbates the"curse of dimensionality,"rendering data from each sample largely disconnected. Additionally, most samples in studies are derived from animal models. There is a substantial difference in epileptogenic mechanism between humans and animals. Establishing a global epilepsy omics database through multi-center collaboration is essential.

Despite these challenges, the ongoing development and optimization of multi-omics technologies, coupled with the adequate training of technical personnel and the reduction in analysis costs, are poised to increasingly improve clinical medicine, particularly in the field of epilepsy.

## Data Availability

No datasets were generated or analysed during the current study.
